# The Hypodermic Use of Sulphate of Quinia

**Published:** 1871-09

**Authors:** Francis L. Haynes

**Affiliations:** One of the Resident Physicians at the Episcopal Hospital


### The Hypodermic Use of the Sulphate of Quinia.

By Francis L. Haynes, M.D., one of the Resident Physicians at the Episcopal Hospital.

We see little concerning this method of administering quinia in the medical periodicals of to-day. Although formerly received with favor, it is now, I think, viewed with distrust by medical men generally. There is, without doubt, good foundation for such a feeling. We have all read or heard alarming accounts of the irritating effects of quinia used in this manner. Thus, Prof. Fonssagrives mentions* two cases in which fatal tetanus followed the hypodermic use of sulphate of quinia dissolved in sulphuric acid and water. Dr. Mitchell, of New Orleans, records† a case in which a deep ulcer, and, finally, fatal tetanus, followed the injection of sulphate of quinia. He does not inform us of the way in which the salt was prepared for use.

It is possible, however, that the bad effects of this method are caused by the large quantity of acid used in dissolving the sulphate, and not by the salt itself. The following experiments and cases tend to support this opinion. They were made upon a healthy adult male.

The first two experiments were made in order to establish stand-

*Lancet, July 6, 1867.

†Abstract of the Medical Sciences, vol. xlvi. p. 100.

ards with which to compare the effects of the subsequent experiments.

*Experiment I.*—One fluidrachm of distilled water was injected over the left biceps muscle of a healthy man. Slight pain, tenderness, redness, and swelling ensued. All these symptoms disappeared in a few hours, except the tenderness, which lasted four days.

*Experiment II.*—One-half a grain of sulphate of quinia was dissolved in acetic acid, and sufficient distilled water added to make half a fluidrachm. This was injected over the left long supinator muscle of the radius. Intense stinging and burning pain attended and followed the injection. On the next day, the skin at the point of injection sloughed to the extent of a patch 9 lines by 3 lines, and one-half of the surface of the forearm became quite red, swollen, tender, and painful. Three days afterwards the pain, swelling, and redness had disappeared. The tenderness persisted until the tenth day. Serum exuded beneath the patch of dead skin, which dried up and formed a thick scab, and which fell off on the thirty-second day after the injection, leaving a depressed cicatrix.

In the succeeding experiments and cases quinia suspended in Bower's glycerine was used. The sulphate of quinia was carefully "rubbed up" with the glycerine (in the proportion of not more than gr. iv. to f℥i). The only inconvenience in the use of this mixture resulted from the fact that the glycerine is apt to dissolve the cement used to unite the different portions of the syringe. When very thick glycerine was used, it was diluted with distilled water.

*Experiment III.*—Injected into the right forearm half a grain of sulphate of quinia suspended in half a fluidrachm of glycerine. The only symptom was very slight pain attending the injection.

*Experiment IV.*—Injected into the same arm, on the same day, one grain of quinia suspended in f℥i glycerine. There was a little more pain, with some redness, which lasted a few minutes. The point of injection remained slightly tender for two days.

*Experiment V.*—Injected into the right forearm gr. ij quinia with f℥i glycerine. There was slight pain for five minutes. The seat of injection became prominent. At the apex of this prominence a livid spot appeared, which was enclosed in a ring of white bloodless skin, surrounding which was a narrow border of red. This persisted for only a few minutes, and was unattended by any disagreeable sensation. The next day there was no redness, pain, or swelling, and tenderness could just be detected.

*Experiment VI.*—Injected four grains of sulphate of quinia suspended in f℥i glycerine. The same appearances as in *Experiment V.* presented themselves. The next day tenderness barely remained.—*Medical Times.*

